# Modeling of Eyld2000-2d Anisotropic Yield Criterion Considering Strength Differential Effect and Analysis of Optimal Calibration Strategy

**DOI:** 10.3390/ma16196445

**Published:** 2023-09-27

**Authors:** Kai Du, Li Dong, Hao Zhang, Zhenkai Mu, Hongrui Dong, Haibo Wang, Yanqiang Ren, Liang Sun, Liang Zhang, Xiaoguang Yuan

**Affiliations:** 1 School of Materials Science and Engineering, Shenyang University of Technology, Shenyang 110870, Chinasunliang324117@163.com (L.S.);; 2 State Key Laboratory of Light Alloy Casting Technology for High-End Equipment, Shenyang 110022, China; 3 Hebei Key Laboratory of Material Near-Net Forming Technology, Hebei University of Science and Technology, Shijiazhuang 050018, China; muzhenk1229@163.com; 4 School of Materials Science and Engineering, Beihang University, Beijing 100191, China; donghongrui@buaa.edu.cn; 5 School of Mechanical and Materials Engineering, North China University of Technology, Beijing 100144, China; wanghaibo@ncut.edu.cn

**Keywords:** sheet metal, yield criterion, strength differential effect, plastic anisotropy, evolving plastic behavior

## Abstract

Sheet metals usually experience various loading paths such as uniaxial tension, uniaxial compression, biaxial tension, and simple shear during the forming process. However, the existing constitutive models cannot always accurately describe blanks’ anisotropic yield and plastic flow behavior of blanks under all typical stress states. Given this, this paper improves the Eyld2000-2d yield criterion by introducing hydrostatic pressure to the A-Eyld2000-2d yield criterion that can describe the strength differential effect of materials. Meanwhile, to control the curvature of the yield surface more effectively, the near-plane strain yield stresses were added in the parameter identification process to calibrate the exponent *m*, so that the exponent is no longer considered as a constant value. Taking the widely used AA6016-T4, AA5754-O, DP980, and QP980 blanks in the automotive stamping industry as an example, the effectiveness of the new model and different parameter identification methods was verified by predicting experimental data under various simple and complex loading paths. Subsequently, the new model employing the optimal parameter identification strategy was compared with four widely used asymmetric yield criteria under associated and non-associated flow rules, including CPB06, LHY2013, S-Y2004, and Hu & Yoon2021, to further verify the accuracy of the proposed constitutive model. The results indicate that parameter identification strategy with variable exponent can significantly improve the flexibility of the yield criterion in describing the plastic anisotropy of blanks. Compared to the other yield criteria examined in this work, the new model provides the best prediction accuracy for the yield stresses and plastic flows of all blanks, especially in the near-plane strain and simple shear stress states. Modeling under the concept of anisotropic hardening can more accurately capture the evolving plastic behavior of blanks than isotropic hardening.

## 1. Introduction

With increasingly strict requirements for lightweight and crashworthiness in the automotive manufacturing industry, an increasing number of aluminum alloy and advanced high-strength steel blanks are widely used [1,2,3,4,5,6]. However, sheet metals usually exhibit serious anisotropic behavior during the forming process and are subjected to various stress states such as uniaxial tension (UT), uniaxial compression (UC), equi-biaxial tension (EBT), near-plane strain (NPS), and simple shear (SS) [7,8]. These complex plastic deformation behaviors pose a grand challenge in the development of high-fidelity constitutive models. Nevertheless, establishing a high-precision constitutive model that can characterize typical stress states and anisotropic behavior is still a main research interest in the field of plastic forming.

To date, researchers have already proposed various anisotropic constitutive models. Hill [9] proposed the famous quadratic anisotropic yield criterion based on the von Mises isotropic yield criterion, which has become one of the most widely used yield criteria due to its simple expression and ease of calculation [10]. Considering the poor ability of the secondary yield criterion to describe the plastic deformation behavior of sheet metals, especially aluminum alloys, Barlat and Lian [11] developed the Barlat89 yield criterion. With the demand for accurate predictions of more mechanical properties, Barlat et al. [12,13] have successively established the yield criteria of Balat94 and Balat96. However, the convexity of the yield criterion cannot be guaranteed, which limits their application. To solve the above issue, Barlat et al. [14] proposed the Yld2000-2d yield criterion applicable to the plane stress state by introducing a fourth-order linear operator to the Cauchy stress tensor. Because Yld2000-2d can describe the anisotropic behavior of blanks more accurately, it has become one of the most widely used advanced yielding criteria in industry and academia. Subsequently, Barlat et al. [15] proposed the Yld2004-18p yield criterion applicable to three-dimensional stress states, which effectively predicted the phenomenon of six or eight earings appearing in deep drawing tests of cylindrical cups for blanks with strong anisotropy. However, due to the large amount of experimental data and complex calculation process required for calibrating parameters, they have not been widely used in industry. Distinguishing from the linear transformation approach, Banabic et al. [16] developed the BBC2005 yield criterion by adding anisotropy parameters in Hershey1954, which can accurately predict the shape of the yield surface. Cazacu and Barlat [17] constructed an orthotropy yield criterion based on the *J*_2_- and *J*_3_-based Drucker frameworks using the theory of the representation of tensor functions, which can accurately describe the plastic anisotropic behaviors of AA6016-T4 and AA2093-T3. Another anisotropic form of the Drucker yield function was introduced through linear transformation tensor [18], which is implemented into Ansys LS-DYNA as *Mat_263 with four ductile fracture criteria developed by the same authors. Meanwhile, to reduce the input of experimental data, Khalfallah et al. [19,20] further proposed a simplified calibration program for the CB2001 yield criterion and verified the effectiveness of the newly developed parameter identification strategy through simulation analysis of cross-shaped deep-drawn cup and tube hydroforming experiments. Lou et al. [21] introduced a reduced Yld2004 function under associate flow rule to model anisotropic plastic behavior both in strength and plastic deformation for spatial and plane stress loading conditions. Recently, Lee et al. [22] coupled quadratic S-Y2009 with non-quadratic Hosford72 and proposed the CQN2017 yield criterion, which can describe the yield stresses anisotropy of blanks under the non-associated flow rule (non-AFR). Inspired by CQN2017, Hu et al. [23] further coupled the fourth-order polynomial yield criterion with the Hosford isotropic yield criterion under the associated flow rule (AFR), which can accurately describe the anisotropic behavior of materials; even for blanks with strong plastic flow anisotropy, it can provide accurate prediction levels. Chen et al. [24] proposed another form of the CQN function by coupling the quadratic S-Y2009 function with the non-quadratic Drucker function to achieve higher computation efficiency with similar accuracy. Hou et al. [25] further replaced the coupling function based on stress components with the coupling function based on stress invariants.

However, most of the yield criteria mentioned above cannot describe the asymmetric yield behavior under tension and compression of materials. Spitzig et al. [26] and Spitzig and Richmond [27] found that the UT yield behavior of aluminum alloys and steels was influenced by superimposed hydrostatic pressure. Therefore, Stoughton and Yoon [28] developed a quadratic asymmetric yield criterion to describe the strength differential (SD) effect of AA2008-T4 and AA2090-T3 aluminum alloys. To seek a yield criterion suitable for describing the anisotropic behavior of HCP structure, Cazacu and Barlat [29] modified the even-form Drucker1949 yield criterion to an odd-form one and proposed the CB2004 yield criterion that uses the invariants of the stress deviator to characterize the asymmetric yield behavior of materials. Subsequently, Cazacu et al. [30] further developed the CPB06 yield criterion containing a fourth-order linear operator. To improve the flexibility of CPB06 in describing plastic anisotropy, Plunkett et al. [31] and Li et al. [32] added additional linear transformation tensors to the CPB06 yield criterion and established CPB06ex2 and M_CPB06 yield criteria. Khan et al. [33] proposed a new method to describe the SD effect by using the Lode angle parameter, which can describe the tension–compression asymmetry individually. Lou et al. [34] modified the Yld2000-2d yield criterion to accurately describe the asymmetric yield behavior of aluminum alloys by introducing the first stress invariant. Yoon et al. [35] and Lou et al. [36] developed two asymmetric yield functions in a form of three stress invariants. Furthermore, Hou et al. [37,38,39] improved the KB93 and Min2016 yield criteria under the non-AFR, which can describe the tension–compression asymmetry of yield stresses and *r-*values, respectively. Hu and Yoon [40] simplified the expression of the Yoon2014 yield criterion by analyzing the transformation tenors on the deviatoric stress invariants, which achieved the analytical calibration of anisotropic parameters while retaining the traditional model’s ability to describe the SD effect. Hu et al. [41] established a more flexible asymmetric yield criterion by reconstructing LHY2013 and verified its accuracy based on FCC and HCP materials. Recently, Lou and Yoon [42] proposed a Lode-dependent asymmetric–anisotropic (LAA) framework by analyzing the correspondence between stress triaxiality and normalized third deviatoric stress invariant under uniaxial and equi-biaxial stress states, which can accurately predict the asymmetric behavior of yield stresses and *r-*values with a 45° increment under uniaxial loading paths.

To accurately describe the dominant plasticity behaviors under SS and NPS stress states, Vegter et al. [43] optimized the exponents of the Yld2000-2d and Yld2004-18p yield criteria based on interpolation methods, which filled the gap in traditional calibration methods for predicting the mechanical properties of SS and NPS stress states. Similarly, Du et al. [44] accurately predicted the normal and diagonal planes yield loci of AA6016-T4, AA5182-O, MP980, and DP490 blanks by incorporating NPS yield stresses along the 0°, 45°, and 90° directions into the calibration of anisotropy parameters and exponents of BBC2008. To address the issue of insufficient accuracy in describing plastic flow under NPS loading paths, Hou et al. [45] further introduced the directions of plastic strain rate along the 0°, 45°, and 90° directions in the NAFR-Poly4 yield criterion to calibrate the anisotropy parameters of the plastic potential function. In addition, it is worth noting that the Yld2000-2d yield criterion can accurately predict the plastic deformation behavior under corresponding stress states when identifying anisotropic parameters through NPS or SS mechanical properties, but it cannot describe both stress states simultaneously [46]. Therefore, He et al. [47] improved the Yld2000-2d yield criterion by introducing a shear-related additional term to enhance the flexibility of the Yld2000-2d, while ensuring the accuracy of describing biaxial tensile (BT) stress states, it can effectively predict anisotropic yield and plastic flow near the SS stress states. Recently, Hu et al. [48] proposed a more flexible Analytical Poly6-18p yield function based on the Analytical Poly6-16p yield criterion, which not only achieved accurate modeling of SS stress along the 45° direction but also incorporated SS stress along the 0° direction into the modeling category.

In summary, considering that existing constitutive models usually cannot accurately describe the anisotropic behavior of blanks under various typical loading conditions such as UT, UC, EBT, NPS, and SS, in this work, the Eyld2000-2d yield criterion was improved to a new model, i.e., the A-Eyld2000-2d yield criterion, that can describe the SD effect by introducing hydrostatic pressure. Meanwhile, to more effectively control the yield locus of the sheet metals, the mathematical constraint that the exponent *m* is a constant value was removed by increasing NPS yield stresses during the parameter calibration process. Through experimental data measured under UT, UC, SS, and BT stress states, the differences in describing the anisotropic yield and plastic flow of AA6016-T4, AA5754-O, DP980, and QP980 using the new model with different parameter identification strategies were systematically evaluated. Subsequently, the new model employing the best parameter identification strategy was compared with four commonly used asymmetric yield criteria, i.e., CPB06, LHY2013, S-Y2004, and Hu & Yoon2021, to further verify the validity and applicability of the new model. Finally, the influence of different hardening concepts on the prediction accuracy of yield criteria in describing the evolving plastic behavior of sheet metals was discussed.

## 2. Modeling Strategies for Describing the Plastic Anisotropic Behavior of Materials under Various Typical Stress States

### 2.1. Asymmetric Eyld2000-2d Yield Criterion

This section improves the Eyld2000-2d yield criterion proposed by He et al. [47] under AFR to describe the SD effect of blanks. The equivalent stress of the newly proposed asymmetric Eyld2000-2d yield criterion (A-Eyld2000-2d) is defined as:

(1) $$\varphi =\bar{\sigma }=\tilde{I}+f^{1/m}$$

(2) $$\tilde{I}=h_{x}\sigma_{11}+h_{y}\sigma_{22}$$

(3) $$f=\frac{1}{2}(f_{1}+f_{2})$$

where φ is the yield function, $\bar{\sigma }$ is the equivalent stress (in this work the rolling direction (RD) was chosen as the reference direction), $\tilde{I}$ is the hydrostatic pressure, *m* is the exponent of the yield criterion, f is the sum of the two isotropic functions $f_{1}$ and $f_{2}$, where $f_{1}$ and $f_{2}$ are expressed as

(4) $$f_{1}=\frac{1}{2}({\left|X_{1}^{\prime }-X_{2}^{\prime }\right|}^{m}+{\left|2X_{1}^{\prime }+X_{2}^{\prime }\right|}^{m}+{\left|X_{1}^{\prime }+2X_{2}^{\prime }\right|}^{m})$$

(5) $$f_{2}=\frac{1}{2}({\left|2X_{1}^{\prime \prime }+X_{2}^{\prime \prime }\right|}^{m}+{\left|X_{1}^{\prime \prime }+2X_{2}^{\prime \prime }\right|}^{m})$$

where $X_{i}^{\prime }$ and $X_{j}^{\prime \prime }$ (i,j=1,2) are the eigenvalues of the stress deviators $X^{\prime }$ and $X^{\prime \prime }$, i.e.,

(6) $$X_{1,2}^{\prime }=\frac{1}{2}(X_{11}^{\prime }+X_{22}^{\prime }\pm \sqrt{{\left(X_{11}^{\prime }-X_{22}^{\prime }\right)}^{2}+4{X_{12}^{\prime }}^{2}})$$

(7) $$X_{1,2}^{\prime \prime }=\frac{1}{2}(X_{11}^{\prime \prime }+X_{22}^{\prime \prime }\pm \sqrt{{\left(X_{11}^{\prime \prime }-X_{22}^{\prime \prime }\right)}^{2}+4{X_{12}^{\prime \prime }}^{2}})$$

where $X_{ij}^{\prime }$ and $X_{ij}^{\prime \prime }$ (i,j=1,2) are obtained through linear transformation of Cauchy stress, there are

(8)  X′=[X11′X22′X12′]=[23C11′−13C12′−13C11′+23C12′023C21′−13C22′−13C21′+23C22′000C′66][σ11σ22σ12]

(9)  X″=[X11″X22″X12″]=[23C11″−13C12″−13C11″+23C12″023C21″−13C22″−13C21″+23C22″000C66″][σ11σ22σ12]

where $C_{ij}^{\prime }$ and $C_{ij}^{\prime \prime }$ (i,j=1,2 or i=j=6) are the anisotropy parameters of the yield criterion, and $\sigma_{ij}$ (i,j=1,2) is the in-plane stress component of the Cauchy stress tensor.

### 2.2. Exponent m Study of A-Eyld2000-2d

**Figure 1:**
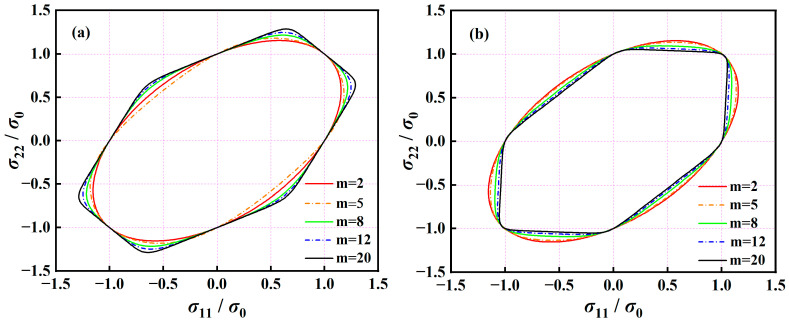
Normalized yield loci of the A-Eyld2000-2d yield criterion calculated using (**a**) isotropic mechanical properties and (**b**) a specific set of anisotropic parameters at different exponents *m*.

To quantitatively clarify the potential regulative effects of the exponent *m* on the curvature of yield locus, the normalized yield loci of the A-Eyld2000-2d yield criterion are calculated using isotropic mechanical properties and a specific set of anisotropic parameters by adjusting the value of *m*, as shown in Figure 1. In general, the uniaxial and equi-biaxial tensile/compressive yield stresses are not affected by changes in the exponent, but the yield points near the NPS and SS stress states exhibit strong exponent sensitivity. When using isotropic mechanical properties, as the exponent increases, the boundary near the SS stress state shrinks first and then expands, while the region corresponding to the NPS stress state continues to expand outward, as shown in Figure 1a. In contrast, when the anisotropy parameters remain constant, as the exponent increases, the normalized yield loci of the NPS and SS stress state regions gradually shrink inward, as shown schematically in Figure 1b. Furthermore, to further understand the prediction capability of the A-Eyld2000-2d yield criterion on plastic anisotropy behavior within the investigated exponent range of *m*∈[2, 20], the ratios PTR and STR of NPS and SS yield stresses to UT yield stress, as defined by Hou et al. [49], are used to quantify the maximum and minimum boundaries described in Figure 1. After calculation, the values of PTR and STR in Figure 1a are 1.2879 and 0.6496, while the values of PTR and STR are 1.0528 and 0.5359 in Figure 1b, respectively, which are sufficient to accurately describe the yield behavior of the vast majority of conventional blanks. The above characteristics provide a theoretical basis for calibrating the exponent and anisotropy parameters of the A-Eyld2000-2d yield criterion using NPS and SS mechanical properties.

### 2.3. Parameter Calibration Programs for A-Eyld2000-2d Yield Criterion

The description ability of the yield criterion is not only related to the modeling expression but also influenced by the calibration methods. Therefore, four different parameter identification strategies are designed for A-Eyld2000-2d in this section to illustrate the regulative effects of the selected mechanical properties and exponent *m* on the curvature of the yield locus. Table 1 lists the mechanical properties employed by different calibration methods, where “√” and “×” represent selected and unselected, respectively. Method #1 is the same as the original Eyld2000-2d yield criterion and does not consider the asymmetric yield behavior of blanks; Method #2 additionally introduces $\sigma_{C0}$ and $\sigma_{C90}$ to calculate $h_{x}$ and $h_{y}$ based on Method #1; Method #3 further adds the NPS yield stresses to identify anisotropy parameters; Method #4 removes the mathematical constraint that the exponent *m* is an integer compared with Method #3. Note that when *m*∈N*, i.e., Methods #1, #2, and #3, *m* is set to 6 for BCC materials and 8 for FCC materials. In addition, it is usually difficult to measure the plastic strain rate *r*_s_ of blanks under SS stress state. Therefore, to avoid the formation of underdetermined equations during calibration process, which leads to non-uniqueness of parameters, in this work, the anisotropy parameters $C_{12}^{\prime \prime }$ and $C_{21}^{\prime \prime }$ related to the normal stress component are made equal.

For different parameter identification strategies, the following minimization objective function is defined to compute the anisotropy parameters.

(10) $$\begin{array}{l} F{\left[C_{11}^{\prime },C_{12}^{\prime },C_{21}^{\prime },C_{22}^{\prime },C_{66}^{\prime },C_{11}^{\prime \prime },C_{12}^{\prime \prime },C_{22}^{\prime \prime },C_{66}^{\prime \prime },h_{x},h_{y},m\right]}_{\mathrm{Min}}=\\ \lambda_{1}\left[\sum_{t=1}^{3}{\left(\frac{\sigma_{Ti}^{\mathrm{cal}}}{\sigma_{Ti}^{\exp}}-1\right)}^{2}\right]+\lambda_{2}\left[\sum_{t=1}^{3}{\left(\frac{r_{Ti}^{\mathrm{cal}}}{r_{Ti}^{\exp}}-1\right)}^{2}\right]+\lambda_{3}{\left(\frac{\sigma_{S45}^{\mathrm{cal}}}{\sigma_{S45}^{\exp}}-1\right)}^{2}\\ +\lambda_{4}\left[{\left(\frac{\sigma_{b}^{\mathrm{cal}}}{\sigma_{b}^{\exp}}-1\right)}^{2}+{\left(\frac{r_{b}^{\mathrm{cal}}}{r_{b}^{\exp}}-1\right)}^{2}\right]\\ +\lambda_{5}\left[{\left(\frac{\sigma_{C0}^{\mathrm{cal}}}{\sigma_{C0}^{\exp}}-1\right)}^{2}+{\left(\frac{\sigma_{C90}^{\mathrm{cal}}}{\sigma_{C90}^{\exp}}-1\right)}^{2}\right]\\ +\lambda_{6}\left\{{\left[\frac{{\left(\sigma_{\mathrm{PS}}^{0}\right)}^{\mathrm{cal}}}{{\left(\sigma_{\mathrm{PS}}^{0}\right)}^{\exp}}-1\right]}^{2}+{\left[\frac{{\left(\sigma_{\mathrm{PS}}^{90}\right)}^{\mathrm{cal}}}{{\left(\sigma_{\mathrm{PS}}^{90}\right)}^{\exp}}-1\right]}^{2}\right\} \end{array}$$

where i = 0, 45, and 90, “cal” and “exp” represent the theoretical calculation values and experimental measurement values, respectively. $\lambda_{j}\left(j=1,2,\ldots ,6\right)$ is the weighting factor, when the corresponding mechanical properties are used in the parameter identification processes λ=1; conversely, λ=0. In this work, the Particle Swarm Optimization function in MATLAB is used to calculate the anisotropy parameters in different calibration methods [37]. To demonstrate the differences in describing plastic anisotropy among several parameter identification strategies, they are applied to AA6016-T4, AA5754-O, DP980, and QP980 blanks, which are widely used in the automotive stamping industry, wherein the mechanical properties of AA6016-T4 are obtained from the reports of Du et al. [50,51], and the mechanical properties of AA5754-O, DP980, and QP980 are extracted from the research of Hou et al. [38].

## 3. Results and Discussions

### 3.1. Prediction Results of Different Parameter Identification Strategies

**Figure 2:**
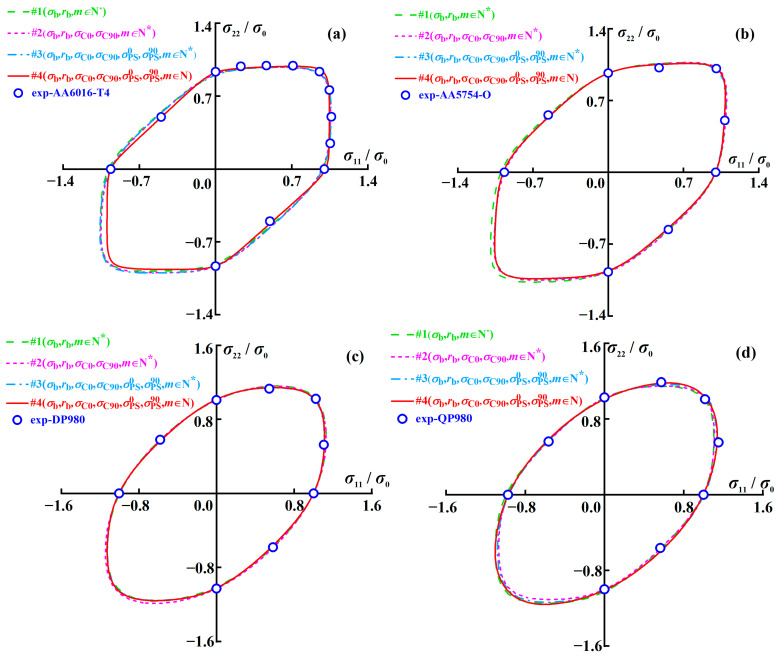
Normalized yield loci of (**a**) AA6016-T4, (**b**) AA5754-O, (**c**) DP980, and (**d**) QP980 blanks predicted by A-Eyld2000-2d yield criterion with different parameter identification strategies.

Figure 2 shows the normalized yield loci of AA6016-T4, AA5754-O, DP980, and QP980 blanks predicted by the A-Eyld2000-2d yield criterion with different parameter identification strategies and compared with the experimental data. Note that the equivalent plastic strains (EPSs) selected for calibrating the anisotropy parameters of four blanks are 0.056, 0.074, 0.050, and 0.060, respectively. These are the maximum EPSs that can be achieved by obtaining experimental data within a uniform deformation range in all loading paths investigated, i.e., UT, UC, EBT, NPS, and SS stress states and different sampling directions. The anisotropy parameters calculated by different parameter identification strategies are summarized in Table A1 in Appendix A. It can be observed that the four parameter identification strategies can reasonably describe the yield loci of four blanks. Compared to Method #1, the other three methods can accurately predict the UC yield stresses, and the ability to describe the SD effect has been improved. This can be attributed to the fact that Methods #2, #3, and #4 employed UC yield stresses during the parameter calibration process, as shown in Table 1. It can also be observed that Methods #1 and #2 exhibit slight deviations in characterizing the NPS stress states, especially for QP980, as shown in Figure 2d. This is because these two methods do not use NPS yield stresses to calibrate the anisotropy parameters, resulting in the inability to more accurately control the curvature of the yield loci. In addition, the four methods can accurately predict the SS yield stresses of AA5754-O, DP980, and QP980 blanks, as shown in Figure 2b–d. However, only Method #4 provides the best prediction accuracy for SS yield stress of AA6016-T4, as shown in Figure 2a. This can be attributed to the exponent of Method #4 having more flexible adjustment space. To further evaluate the ability of the A-Eyld2000-2d yield criterion with different parameter identification strategies in describing yield loci, the errors are calculated through the analytical indicator used by Du et al. [52]:

(11) $$\Delta_{\mathrm{yl}}=\sqrt{\frac{1}{N}\sum_{t=1}^{N}{\left\{\frac{f\left[{\left(\sigma_{ij}\right)}^{\exp}\left(t\right)\right]}{\bar{\sigma }}-1\right\}}^{2}}$$

where $\sigma_{ij}$ (*i*, *j* = 1, 2) is the experimental stress component, *N* is the number of loading paths, *t* is the *t*^th^ loading path.

**Figure 3:**
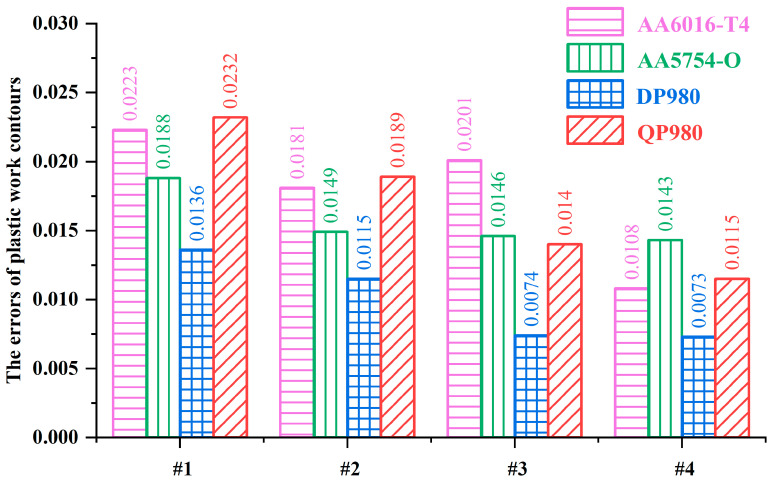
Prediction errors of normalized yield loci of AA6016-T4, AA5754-O, DP980, and QP980 blanks calculated by the A-Eyld2000-2d yield criterion with different parameter identification strategies.

Figure 3 shows the prediction errors of the normalized yield loci calculated by the A-Eyld2000-2d yield criterion with different parameter identification strategies for AA6016-T4, AA5754-O, DP980, and QP980 blanks. It can be observed that Method #4 provides the best prediction accuracy for these four blanks, while Method #1 provides the highest level of prediction errors. Compared with Method #1, the errors of AA6016-T4, AA5754-O, DP980, and QP980 blanks predicted by Method #4 decreased by 51.57%, 23.94%, 46.32%, and 50.43%, respectively. Methods #3 and #4 are compared to further illustrate the contribution of exponent *m* to improving the accuracy of yield criteria in describing the yield locus of blanks, which have great accuracy and insignificant difference in describing the mechanical properties of DP980 and QP980 blanks under SS and NPS stress states, as shown in Figure 2c,d. This can be attributed to Method #4 removing the restriction of the integer exponent of the yield criterion, and thereby enhancing the ability of the yield criterion to adjust the curvature of the yield locus. Meanwhile, the quantitative analysis of errors showed that compared with Method #3, the errors of AA6016-T4, AA5754-O, DP980, and QP980 blanks predicted by Method #4 decreased by 46.27%, 2.05%, 1.35%, and 17.86%, respectively, as shown in Figure 3. This proves that changing the exponent from integer to non-integer has a positive effect on improving the prediction accuracy of the yield criterion. Note that DP980 always has the lowest prediction errors for the four engineering materials investigated. This is because there is no phenomenon of residual austenite transforming into martensite in DP980 compared with QP980 [41]. Meanwhile, the shapes of the yield loci of DP980 are more rounded than that of aluminum alloy blanks. Therefore, regardless of the parameter calibration method used, it will always provide the best prediction accuracy for the DP980.

**Figure 4:**
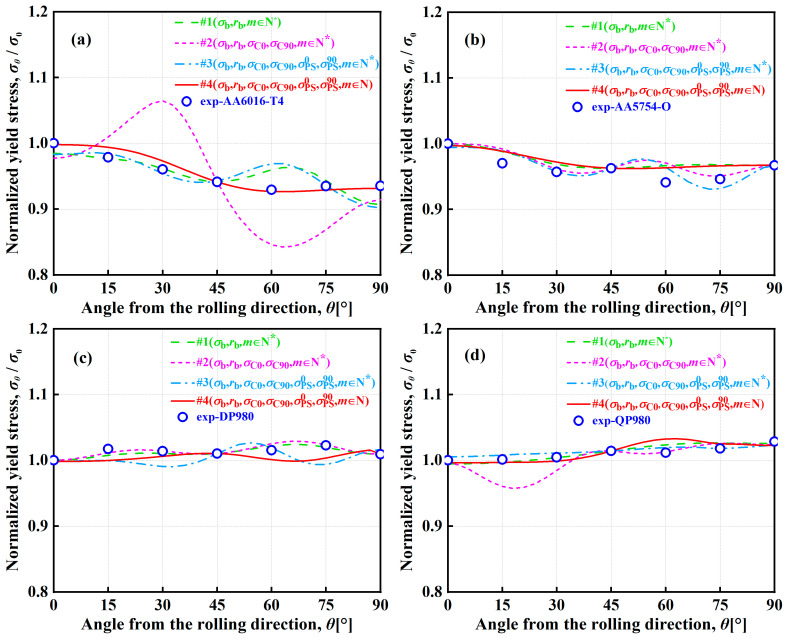
Normalized UT yield stresses of (**a**) AA6016-T4, (**b**) AA5754-O, (**c**) DP980, and (**d**) QP980 blanks predicted by the A-Eyld2000-2d yield criterion with different parameter identification strategies.

**Figure 5:**
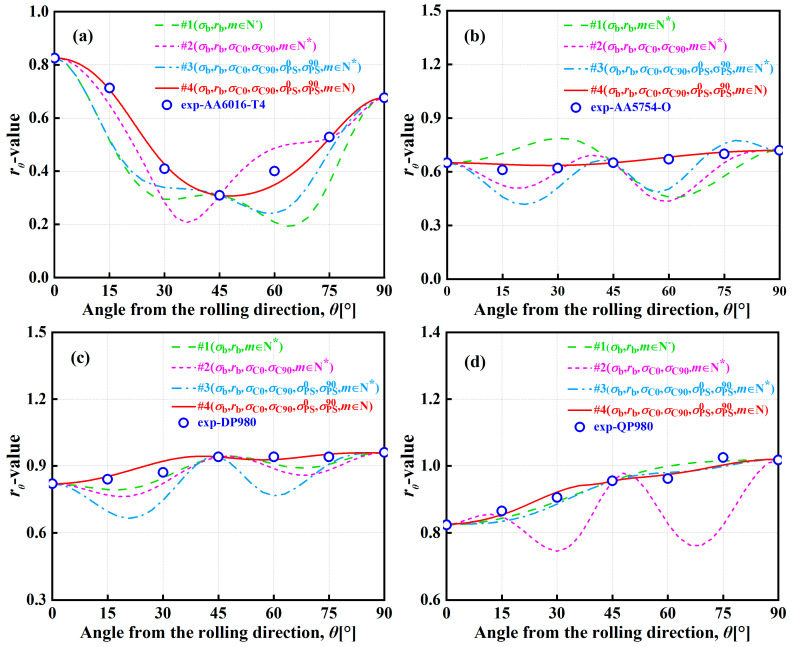
*r*-values of (**a**) AA6016-T4, (**b**) AA5754-O, (**c**) DP980, and (**d**) QP980 blanks predicted by the A-Eyld2000-2d yield criterion with different parameter identification strategies.

Figure 4 and Figure 5 show the normalized UT yield stresses and *r*-values of AA6016-T4, AA5754-O, DP980, and QP980 blanks predicted by the A-Eyld2000-2d yield criteria with different parameter identification strategies, respectively. It can be observed that the four parameter identification strategies can accurately predict the normalized UT yield stresses and *r*-values along the RD, diagonal direction (DD), and transverse direction (TD), but there are certain deviations when describing the normalized UT yield stresses and *r*-values at 15°, 30°, 60°, and 75° directions along the RD. This can be attributed to the fact that these four methods employ mechanical properties along the RD, DD, and TD to identify anisotropy parameters. Therefore, when predicting normalized yield stress and *r*-values along the RD, DD, and TD, it is more accurate than other angles. However, it is worth noting that Method #2 has obvious errors in describing the UT yield stresses of blanks, especially for AA6016-T4 and QP980, as shown in Figure 4a,d. Meanwhile, Method #4 always provides the best prediction accuracy for in-plane anisotropic behavior of *r*-values, which is the most evident in AA6016-T4 and AA5754-O, i.e., the prediction results provided by Methods #1, #2, and #3 deviate significantly from the experimental values, as shown in Figure 5a,b. To further evaluate the ability of the A-Eyld2000-2d yield criterion with different parameter identification strategies to describe normalized UT yield stresses and *r*-values, the analysis indicators proposed by Stoughton and Yoon [53] are used to calculate the prediction errors $\Delta_{\mathrm{UT}}$ and $\Delta_{r}$:

(12) $$\Delta_{\mathrm{UT}}=\sqrt{\frac{1}{12}\left[\begin{array}{l} {\left(\frac{\sigma_{0}^{\mathrm{cal}}}{\sigma_{0}^{\exp}}-1\right)}^{2}+2{\left(\frac{\sigma_{15}^{\mathrm{cal}}}{\sigma_{15}^{\exp}}-1\right)}^{2}+\\ 2{\left(\frac{\sigma_{30}^{\mathrm{cal}}}{\sigma_{30}^{\exp}}-1\right)}^{2}+2{\left(\frac{\sigma_{45}^{\mathrm{cal}}}{\sigma_{45}^{\exp}}-1\right)}^{2}+2{\left(\frac{\sigma_{60}^{\mathrm{cal}}}{\sigma_{60}^{\exp}}-1\right)}^{2}\\ +2{\left(\frac{\sigma_{75}^{\mathrm{cal}}}{\sigma_{75}^{\exp}}-1\right)}^{2}+{\left(\frac{\sigma_{90}^{\mathrm{cal}}}{\sigma_{90}^{\exp}}-1\right)}^{2} \end{array}\right]}$$

(13) $$\Delta_{r_{}}=\sqrt{\frac{1}{12}\left[\begin{array}{l} {\left(\frac{r_{0}^{\mathrm{cal}}}{r_{0}^{\exp}}-1\right)}^{2}+2{\left(\frac{r_{15}^{\mathrm{cal}}}{r_{15}^{\exp}}-1\right)}^{2}+\\ 2{\left(\frac{r_{30}^{\mathrm{cal}}}{r_{30}^{\exp}}-1\right)}^{2}+2{\left(\frac{r_{45}^{\mathrm{cal}}}{r_{45}^{\exp}}-1\right)}^{2}+2{\left(\frac{r_{60}^{\mathrm{cal}}}{r_{60}^{\exp}}-1\right)}^{2}\\ +2{\left(\frac{r_{75}^{\mathrm{cal}}}{r_{75}^{\exp}}-1\right)}^{2}+{\left(\frac{r_{90}^{\mathrm{cal}}}{r_{90}^{\exp}}-1\right)}^{2} \end{array}\right]}$$

Due to the significant anisotropic behavior in the 105°, 120°, 135°, 150°, and 165° directions along the RD, the weights of yield stresses and *r*-values under the UT stress states in the 15°, 30°, 45°, 60°, and 75° directions are increased.

Table 2 lists the errors of the normalized UT yield stress and *r*-values predicted by the A-Eyld2000-2d yield criterion with different parameter identification strategies. The average errors of *r*-values provided by the four methods for all engineering materials investigated are 0.0911, 0.1229, 0.1294, and 0.0293, respectively. Obviously, compared with Methods #1, #2, and #3, Method #4 provides the minimum $\Delta_{r}$ for these four blanks, and the average errors are reduced by 67.87%, 76.19%, and 77.40%, respectively. The average errors of the four methods in predicting UT yield stresses are 0.0114, 0.0265, 0.0147, and 0.0121, respectively. This indicates that except for Method #2, the prediction accuracy of the other methods is not significantly different. In summary, it can be concluded that among all the calibration methods investigated, Method #4 is the best and can accurately describe the plastic anisotropy behavior of blanks under UT, UC, SS, NPS, and EBT stress states, especially for the plastic flow anisotropic behavior.

### 3.2. Prediction Results of Several Asymmetric Yield Criteria

To further present the advancement of the new model, the A-Eyld2000-2d yield criterion employing the best parameter identification strategy is compared with several asymmetric yield criteria, CPB06 [30], LHY2013 [34], S-Y2004 [28], and Hu & Yoon2021 [40], which are widely used under the AFR and non-AFR. Combining quantitative analysis of errors, a systematic evaluation is made for the describing ability of different asymmetric yield criteria to the anisotropic behavior of four blanks.

Table 3 lists the mechanical properties required to calibrate anisotropic parameters for several asymmetric yield criteria. Note that the yield models S-Y2004 and Hu & Yoon2021 under the non-AFR use S-Y2009 as the plastic potential function (PPF). The anisotropy parameters calculated by several asymmetric yield criteria are summarized in Table A2 and Table A3 in Appendix B.

**Figure 6:**
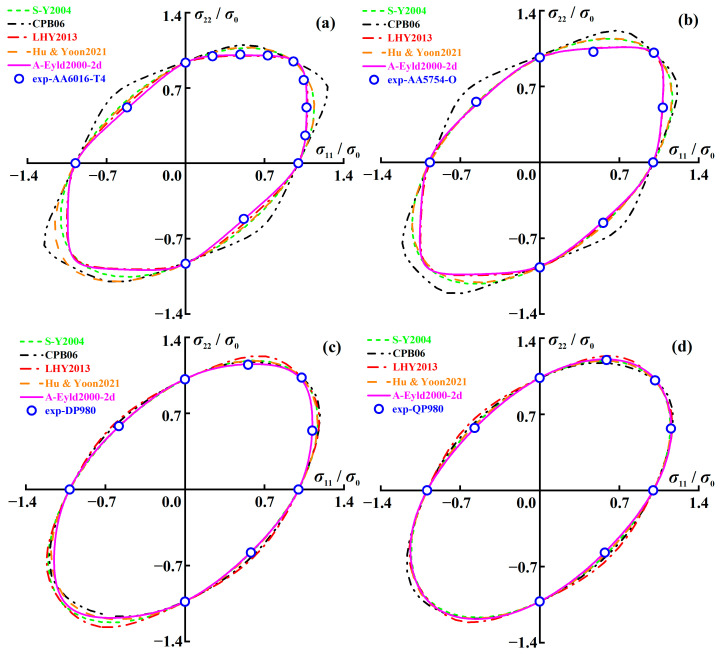
Normalized yield loci of (**a**) AA6016-T4, (**b**) AA5754-O, (**c**) DP980, and (**d**) QP980 blanks predicted by several asymmetric yield criteria.

Figure 6 shows the normalized yield locus of AA6016-T4, AA5754-O, DP980, and QP980 blanks predicted by several asymmetric yield criteria. CPB06 shows significant deviation when describing the yield loci of AA6016-T4 and AA5754-O, as shown in Figure 6a,b. However, for DP980 and QP980, the errors of normalized yield loci predicted by the five asymmetric yield criteria are relatively small, and the differences are mainly reflected in the SS and NPS stress states, as shown in Figure 6c,d. In addition, it can also be observed that S-Y2004 and Hu & Yoon2021 have very similar abilities to describe the normalized yield loci of four blanks, and their prediction accuracy is always better than CPB06 under NPS and SS stress states. To further quantitatively evaluate the ability of five asymmetric yield criteria to describe the normalized yield loci of four blanks, the errors between the normalized yield loci and experimental data are calculated by Equation (11).

**Figure 7:**
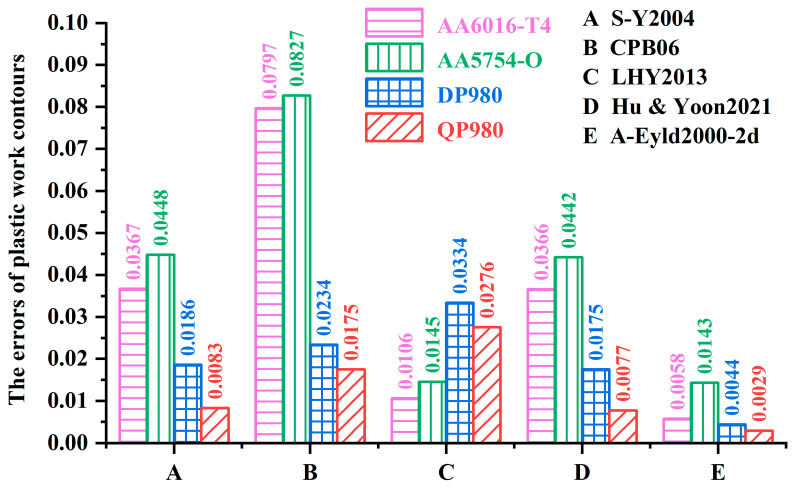
Prediction errors of normalized yield loci of AA6016-T4, AA5754-O, DP980, and QP980 calculated by several asymmetric yield criteria.

Figure 7 shows the errors of normalized yield loci of AA6016-T4, AA5754-O, DP980, and QP980 blanks calculated by several asymmetric yield criteria. The average errors of the five yield criteria for the four engineering materials investigated are 0.0271, 0.0508, 0.0215, 0.0265, and 0.0069, respectively. It can be observed that A-Eyld2000-2d can provide the minimum prediction errors for the normalized yield loci of these four blanks, especially for NPS and SS stress states, which can be attributed to the new model using more comprehensive stress state data to calibrate the anisotropy parameters and exponent *m*. In addition, CPB06 provides the highest errors for AA6016-T4 and AA5754-O. Compared to A-Eyld2000-2d, the errors of both blanks have increased by 92.72% and 82.71%, respectively, while LHY2013 always has lower errors than S-Y2004 and Hu & Yoon2021, and the average errors are reduced by 20.66% and 18.87%, respectively. However, LHY2013 provides the worst prediction accuracy for DP980 and QP980, which is inconsistent with conventional cognition that increasing the number of yield stresses to identify anisotropy parameters usually helps to improve the prediction accuracy of yield criteria in describing yield loci directly related to stress. This can be attributed to the high dependence on blanks, i.e., among the four engineering materials investigated in this study, the CPB06 yield criterion is more suitable for describing the yield loci of BCC materials, while the LHY2013 yield criterion shows higher applicability to FCC materials.

**Figure 8:**
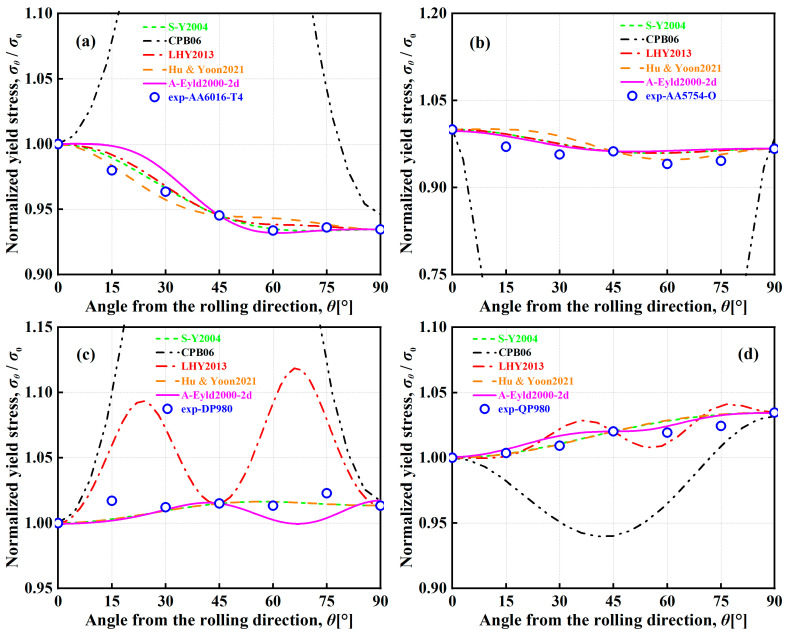
Normalized UT yield stresses of (**a**) AA6016-T4, (**b**) AA5754-O, (**c**) DP980, and (**d**) QP980 predicted by several asymmetric yield criteria.

**Figure 9:**
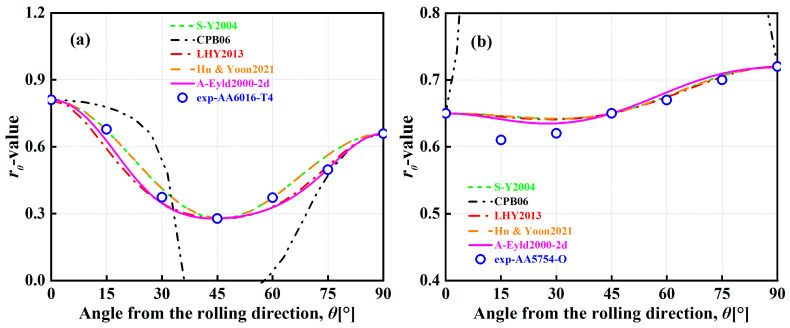

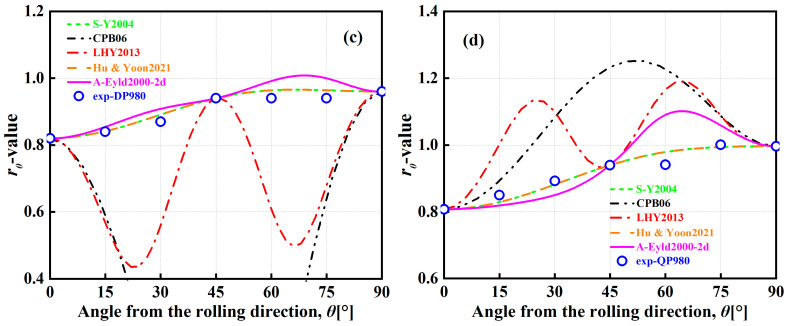
*r*-values of (**a**) AA6016-T4, (**b**) AA5754-O, (**c**) DP980, and (**d**) QP980 predicted by several asymmetric yield criteria.

Figure 8 and Figure 9 show the normalized UT yield stresses and *r*-values of AA6016-T4, AA5754-O, DP980, and QP980 blanks predicted by several asymmetric yield criteria, respectively. It can be observed that the curves of CPB06 in characterizing the in-plane anisotropic behaviors of four blanks show extremely obvious and strong upward convex or downward concave trends. The curves deviate significantly from the experimental values of UT yield stresses for these four blanks. Because the CPB06 yield criterion does not use the mechanical properties along the DD to identify anisotropy parameters, it exhibits drastic fluctuations in predicting the normalized UT yield stresses, as shown in Figure 8 and Figure 9. This indicates that the prerequisite for reasonably predicting the UT mechanical properties of blanks through the yield criterion is to ensure the priority of calibration along the RD, DD, and TD. However, LHY2013 has large errors in predicting the normalized UT yield stresses and *r*-values of DP980 and QP980, and the curves show a relatively obvious fluctuation. Except for CPB06 and LHY2013, the other three yield criteria can predict the in-plane anisotropic behaviors of four blanks more reasonably. The prediction curves of S-Y2004 and Hu & Yoon2021 have the smallest variations and show the weakest fluctuations. Furthermore, although S-Y2004, Hu & Yoon2021, and A-Eyld2000-2d show different trends in predicting the normalized UT yield stresses, they all show good accuracy in predicting the normalized UT yield stresses and *r*-values of four blanks along the RD, DD, and TD. To further quantitatively evaluate the ability of the five yield criteria to predict the normalized UT yield stresses and *r*-values of four blanks, the errors $\Delta_{\mathrm{UT}}$ and $\Delta_{r}$ of several asymmetric yield criteria to predict the normalized UT yield stresses and *r*-values are calculated by Equations (12) and (13), respectively.

Table 4 shows the errors $\Delta_{\mathrm{UT}}$ and $\Delta_{r}$ for predicting the normalized UT yield stresses of four blanks based on several asymmetric yield criteria. The average errors of the normalized UT yield stresses predicted by the five yield criteria for the four engineering materials investigated are 0.0082, 0.3323, 0.0201, 0.0092, and 0.0107, respectively, and the average errors of *r*-values are 0.0339, 1.6855, 0.1331, 0.0339, and 0.0504, respectively. In general, CPB06 always provides the highest errors. Compared with the other four yield criteria, the average errors of CPB06 in describing normalized UT yield stresses increased by 97.53%, 93.95%, 97.23%, and 96.78%, respectively. The average errors in describing *r*-values increased by 97.99%, 92.10%, 97.99%, and 97.01%, respectively, while the quadratic yield function S-Y2004 achieves the best returns in predicting normalized UT yield stresses. Due to the application of the same PPF in S-Y2004 and Hu & Yoon2021, the calculated $\Delta_{r}$ are completely equal. In summary, compared with other asymmetric yield criteria, A-Eyld2000-2d can always make the most accurate prediction of the normalized yield loci of four blanks, especially under NPS and SS stress states. Considering that the blanks are mainly subjected to multi-axial stress states in the actual forming process, and none of the yield criteria can accurately predict the plastic anisotropy behavior under all loading paths, although A-Eyld2000-2d is not stable enough and there exists a certain deviation to describing the normalized UT yield stresses and *r*-values, as shown in Figure 8a–c and Figure 9c,d, it is still the best asymmetric yield criteria investigated.

### 3.3. Evolving Plastic Behavior

The deformation characteristics of sheet metals under a specific level of EPS cannot fully reflect the anisotropic behavior during the forming process [54], i.e., the materials exhibit an anisotropic hardening (AH) phenomenon. Therefore, this section further describes the yield loci, UT yield stresses, and *r*-values of four blanks at different EPSs and compared with the predicted results of the isotropic hardening (IH) concept.

**Figure 10:**
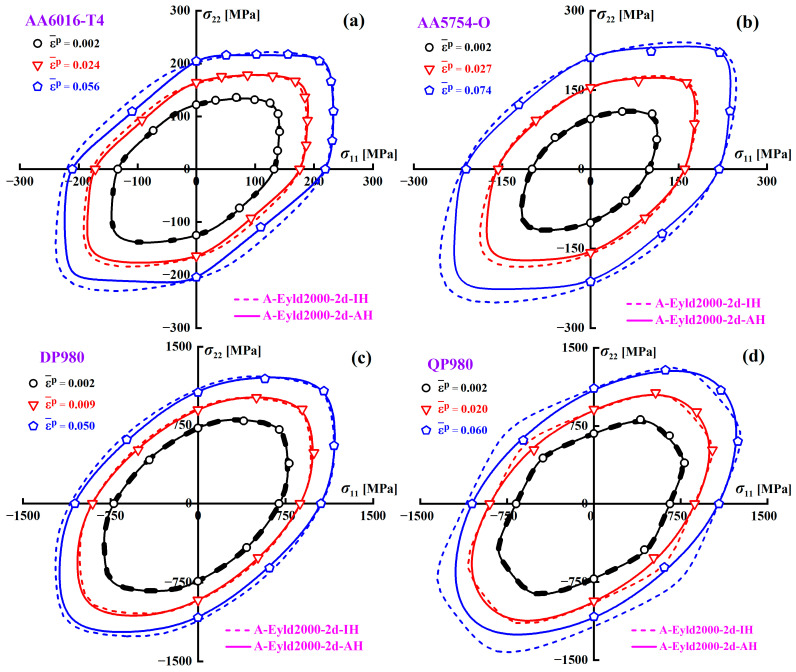
Yield loci of (**a**) AA6016-T4, (**b**) AA5754-O, (**c**) DP980, and (**d**) QP980 blanks predicted by IH and AH concepts at various EPSs.

Figure 10 shows the yield loci of AA6016-T4, AA5754-O, DP980, and QP980 blanks predicted by A-Eyld2000-2d based on the IH and AH concepts at various EPSs. Whether applying IH or AH concepts, both can accurately predict the initial yield loci of four blanks at EPS = 0.002. However, the IH concept exhibits obvious defects in describing the yield loci of four blanks with the increase of EPS. Overall, the SS yield stress and UC yield stress along the RD predicted by the IH concept for these four blanks have significant errors compared to experimental data, especially in the stage of large plastic strain. In addition, the UC yield stresses along the TD of AA5754-O, DP980, and QP980 predicted by the IH concept also have obvious errors, as shown in Figure 10b–d. Furthermore, it can be observed that the IH concept is difficult to provide accurate prediction results for AA5754-O and QP980 under NPS and EBT stress states, as shown in Figure 10b,d. In contrast, the AH concept can always accurately predict the yield loci of four blanks with changes in EPS. This can be attributed to the AH concept using instantaneous mechanical properties to identify the anisotropy parameters of the yield criterion. In other words, the IH concept cannot accurately predict the yield loci under all EPS conditions, which is most significant in AA6016-T4 and QP980, as shown in Figure 10a,d.

**Figure 11:**
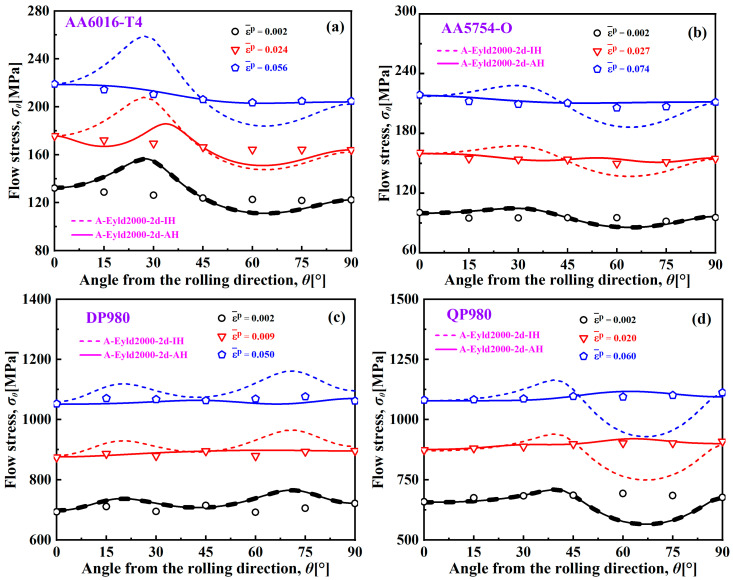
UT yield stresses of (**a**) AA6016-T4, (**b**) AA5754-O, (**c**) DP980, and (**d**) QP980 predicted by IH and AH concepts at various EPSs.

Figure 11 shows the UT yield stresses of AA6016-T4, AA5754-O, DP980, and QP980 predicted by the A-Eyld2000-2d yield criterion based on the IH and AH concepts at various EPSs. For AA6016-T4 and QP980, because the curve shapes of UT yield stress predicted by the IH concept are calculated through the anisotropic parameters at the initial yield point, the prediction accuracy gradually deteriorates with the increase of equivalent plastic strain, while the accuracy of UT curves predicted based on the AH concept gradually improves, as shown in Figure 11a,d. Meanwhile, it can also be observed that the curve obtained based on the AH concept is more stable with the continuous increase of EPS, which makes the prediction accuracy of UT yield stresses at 15° intervals along the RD much higher than that of the IH concept. This indicates that the prediction effect of the A-Eyld2000-2d yield criterion based on the AH concept on the UT yield stresses of four blanks become increasingly excellent.

**Figure 12:**
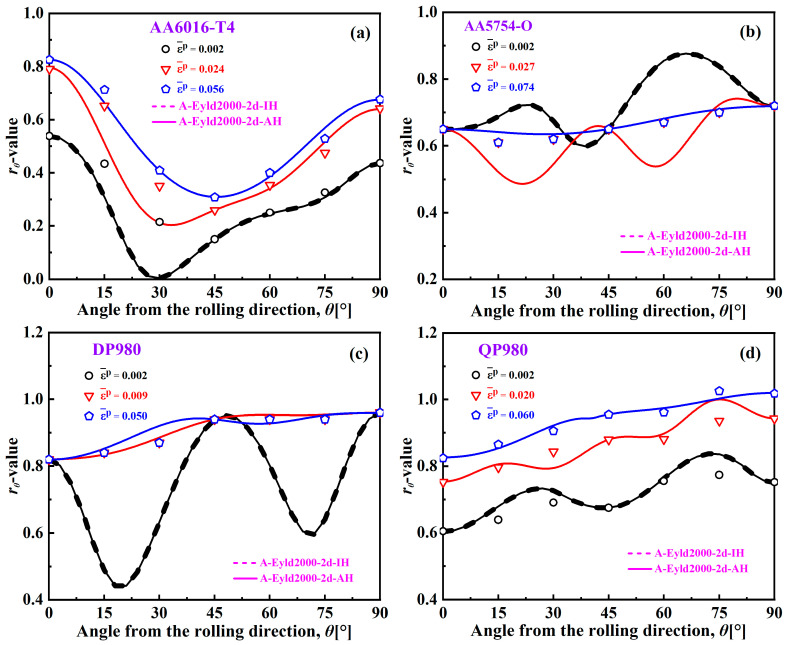
*r*-values of (**a**) AA6016-T4, (**b**) AA5754-O, (**c**) DP980, and (**d**) QP980 blanks predicted by IH and AH concepts at various EPSs.

Figure 12 shows the *r-*values of AA6016-T4, AA5754-O, DP980, and QP980 predicted by the A-Eyld2000-2d yield criterion based on the IH and AH concepts at various EPSs. These two different concepts have the same ability to describe the *r*-values at the initial yield point for these four blanks, and can accurately predict *r*-values along the RD, DD, and TD. However, the IH concept is difficult to provide reasonable predictions for the *r*-values of AA6016-T4 and QP980 with the EPS increases. In contrast, the AH concept can effectively describe the *r*-values along the RD, DD, and TD, and the prediction accuracy of *r*-values in other directions has also become increasingly accurate, as shown in Figure 12a–d. In addition, although there is no evolving flow behavior in the *r*-values of AA5754-O and DP980, the ability of the AH concept to describe *r*-values at 15° intervals along the RD is also becoming stronger with the increase of EPS, as shown in Figure 12b,c. This indicates that it is necessary to describe the evolving plastic behavior of blanks through the AH concept.

## 4. Conclusions

In this work, the Eyld2000-2d yield criterion was improved by introducing hydrostatic pressure and proposed an A-Eyld2000-2d asymmetric yield criterion that can describe the SD effect and anisotropic hardening. Four different parameter identification strategies were designed for the new model, and their validity and applicability in describing plastic anisotropy were verified through AA6016-T4, AA5754-O, DP980, and QP980 blanks. Meanwhile, the A-Eyld2000-2d employing the optimal parameter identification strategy was compared with several existing pressure-sensitive asymmetric yield criteria under AFR and non-AFR. Finally, the influence of different hardening concepts on the evolving plastic behavior of blanks was analyzed, and the following conclusions can be drawn:

(1) The A-Eyld2000-2d yield criterion proposed by coupling hydrostatic pressure method has higher prediction accuracy for the tensile and compressive asymmetric yield behavior of sheet metals compared to the traditional Eyld2000-2d yield criterion.
(2) It is feasible to introduce additional NPS yield stresses to calibrate the exponent *m* of the new model. The parameter identification strategy with a non-integer exponent can significantly improve the prediction ability of the yield criterion for plastic anisotropic behavior, especially for in-plane plastic flow.
(3) The A-Eyld2000-2d yield criterion can provide the best prediction accuracy for the yield loci of four blanks, compared to the S-Y2004, CPB06, LHY2013, and Hu & Yoon2021 yield criteria. The ability to describe UT yield stresses and *r*-values also shows reasonable prediction accuracy, which is most suitable for characterizing the plastic anisotropy behavior of these four blanks.
(4) The deformation characteristics of the initial yield point cannot fully reflect the plastic anisotropic behavior of blanks during the entire deformation stage, considering that the anisotropic hardening concept can significantly improve the ability of the yield criterion to describe the evolving yield behavior and provide sufficient power for the accurate characterization of anisotropic behavior at various EPSs.

## Figures and Tables

**Table 1:** Mechanical properties required for different parameter identification strategies of the A-Eyld2000-2d yield criterion.

Mechanical Properties		Parameter Identification Strategies							
σ	r	#1 *m*∈N*		#2 *m*∈N*		#3 m∈N*		#4 *m*∈N	
$\sigma_{T0}$	$r_{T0}$	√	√	√	√	√	√	√	√
$\sigma_{T45}$	$r_{T45}$	√	√	√	√	√	√	√	√
$\sigma_{T90}$	$r_{T90}$	√	√	√	√	√	√	√	√
$\sigma_{b}$	$r_{b}$	√	√	√	√	√	√	√	√
$\sigma_{C0}$	-	×	-	√	-	√	-	√	-
$\sigma_{C90}$	-	×	-	√	-	√	-	√	-
$\sigma_{S45}$	-	√	-	√	-	√	-	√	-
$\sigma_{\mathrm{PS}}^{0}$	-	×	-	×	-	√	-	√	-
$\sigma_{\mathrm{PS}}^{90}$	-	×	-	×	-	√	-	√	-

**Table 2:** Errors of the normalized UT yield stress and *r*-values predicted by the A-Eyld2000-2d yield criterion with different parameter identification strategies.

Methods	AA6016-T4		AA5754-O		DP980		QP980	
	$\Delta_{UT}$	$\Delta_{r}$	$\Delta_{UT}$	$\Delta_{r_{}}$	$\Delta_{UT}$	$\Delta_{r_{}}$	$\Delta_{UT}$	$\Delta_{r_{}}$
#1	0.0170	0.2865	0.0172	0.0254	0.0051	0.0324	0.0064	0.0199
#2	0.0662	0.1590	0.0160	0.1573	0.0047	0.0542	0.0190	0.1209
#3	0.0211	0.2133	0.0145	0.1642	0.0166	0.1196	0.0066	0.0206
#4	0.0089	0.0553	0.0165	0.0247	0.0124	0.0255	0.0104	0.0115

**Table 3:** Mechanical properties required to calibrate anisotropic parameters for several asymmetric yield criteria.

Mechanical Properties		Asymmetric Yield Criteria									
$\sigma_{}$	r	AFR						NAFR			
		CPB06		LHY2013		A-Eyld2000-2d		S-Y2004		Hu &Yoon2021	
$\sigma_{T0}$	$r_{T0}$	√	√	√	√	√	√	√	×	√	×
$\sigma_{T45}$	$r_{T45}$	×	×	√	√	√	√	√	×	√	×
$\sigma_{T90}$	$r_{T90}$	√	√	√	√	√	√	√	×	√	×
$\sigma_{b}$	$r_{b}$	√	×	√	√	√	√	√	×	√	×
$\sigma_{C0}$	-	√	-	√	-	√	-	√	-	√	-
$\sigma_{C45}$	-	×	-	×	-	×	-	×	-	√	-
$\sigma_{C90}$	-	√	-	√	-	√	-	√	-	√	-
$\sigma_{Cb}$	-	×	-	×	-	×	-	×	-	√	-
$\sigma_{S45}$	-	×	-	×	-	√	-	×	-	×	-
$\sigma_{PS}^{0}$	-	×	-	×	-	√	-	×	-	×	-
$\sigma_{PS}^{90}$	-	×	-	×	-	√	-	×	-	×	-

**Table 4:** Errors of normalized UT yield stresses and *r*-values predicted by several asymmetric yield criteria.

Yield Criteria	AA6016-T4		AA5754-O		DP980		QP980	
	$\Delta_{UT}$	$\Delta_{r_{}}$	$\Delta_{UT}$	$\Delta_{r_{}}$	$\Delta_{UT}$	$\Delta_{r}$	$\Delta_{UT}$	$\Delta_{r_{}}$
S-Y2004	0.0044	0.0689	0.0166	0.0287	0.0067	0.0178	0.0051	0.0201
CPB06	0.3354	0.8146	0.5127	4.8851	0.4343	0.8401	0.0466	0.2023
LHY2013	0.0058	0.0769	0.0166	0.0275	0.0490	0.2692	0.0090	0.1588
Hu & Yoon2021	0.0053	0.0689	0.0193	0.0287	0.0068	0.0178	0.0052	0.0201
A-Eyld2000-2d	0.0103	0.0645	0.0165	0.0247	0.0107	0.0393	0.0051	0.0730

## Data Availability

The data that support the findings of this study are available from the corresponding authors upon reasonable request.

## Appendix A. Anisotropy Parameters Calculated by Different Parameter Identification Strategies

**Table A1:** Anisotropy parameters calculation results of AA6016-T4, AA5754-O, DP980, and QP980 blanks.

Materials	Methods	Anisotropy Parameters											
		$C_{11}^{\prime }$	$C_{12}^{\prime }$	$C_{21}^{\prime }$	$C_{22}^{\prime }$	$C_{66}^{\prime }$	$C_{11}^{\prime \prime }$	$C_{12}^{\prime \prime }$	$C_{22}^{\prime \prime }$	$C_{66}^{\prime \prime }$	$h_{x}$	$h_{y}$	m
AA6016-T4	#1	0.0639	1.2217	−1.0993	−1.1564	−0.6901	0.0240	−0.9873	0.0670	−1.5581	-	-	8
	#2	0.0540	1.1636	1.0995	−0.0462	0.9001	0.1775	0.3873	0.8453	−1.5309	−0.0076	0.0116	8
	#3	−0.0491	−1.2111	−1.0499	0.0423	−0.8238	0.0056	−0.9575	0.1264	1.5649	−0.0066	0.0136	8
	#4	1.0829	1.0797	−0.0457	−1.1160	0.9356	0.0202	−0.8239	−0.3891	1.2707	−0.0235	0.0021	17.3657
AA5754-O	#1	−0.9159	0.0657	0.0588	−1.0801	1.0802	−0.0689	−1.0264	0.0650	−1.2148	-	-	8
	#2	0.9543	−0.1929	−0.9290	−0.7634	−1.0551	−1.0760	0.0305	−1.1241	1.2151	−0.0171	0.0021	8
	#3	−0.9956	−1.0216	0.9945	−0.0689	−1.0386	−0.0486	1.0663	−0.0795	1.2319	−0.0088	−0.0046	8
	#4	−0.9972	0.0456	−0.0092	−1.1091	1.0798	−1.0682	0.0736	−1.0055	1.2479	−0.0101	−0.0049	8.4664
DP980	#1	−0.0499	−1.0020	−1.0562	−0.0830	1.0854	0.6179	−1.1072	0.5978	−1.2492	-	-	6
	#2	−1.0467	−0.0945	−0.0527	−0.9848	1.0894	−0.6309	1.1125	−0.6240	1.1477	0.0019	0.0092	6
	#3	1.1080	−0.0161	−1.1077	−1.0329	−1.0674	0.1468	−0.7867	−0.1393	−0.9006	0.0046	0.0027	6
	#4	−0.0183	1.0318	1.1271	0.0186	1.0985	0.1808	−0.5419	−0.4794	−0.8793	0.0047	0.0028	6.0340
QP980	#1	−1.1328	−0.0153	0.0074	−1.0673	−1.1022	0.2161	−0.7535	0.0491	0.6944	-	-	6
	#2	0.0104	−1.1038	1.0940	1.1144	1.0678	−0.7402	−0.2993	−0.1210	1.0231	−0.0109	−0.0110	6
	#3	−1.1117	−0.0749	−0.0059	−1.0243	−1.0820	−0.5555	1.0464	−0.5353	1.3200	−0.0188	−0.0014	6
	#4	−1.0697	0.2114	−0.2727	−0.8849	1.2262	0.3140	−0.9775	−0.2876	−0.8423	−0.0123	−0.0019	2.8502

## Appendix B. Anisotropy Parameters Calculated by Different Yield Criteria

**Table A2:** Anisotropy parameters of AA6016-T4 and AA5754-O calculated by different yield criteria.

Yield Criteria	AA6016-T4		AA5754-O	
S-Y2004	*α*_1_	−7 × 10^−5^	*α*_1_	−8 × 10^−5^
	*α*_2_	−3 × 10^−6^	*α*_2_	4 × 10^−5^
	*λ_y_*_1_	1.0274	*λ_y_*_1_	1.0279
	*λ_y_*_2_	1.1466	*λ_y_*_2_	1.0605
	*ν_y_*	0.5199	*ν_y_*	0.5586
	*ρ_y_*	1.7016	*ρ_y_*	1.7021
CPB06	$C_{11}$	0.0089	$C_{11}$	−3 × 10^−6^
	$C_{22}$	−0.0012	$C_{22}$	0.0003
	$C_{33}$	−0.0012	$C_{33}$	0.0075
	$C_{12}$	0.0006	$C_{12}$	0.0085
	$C_{13}$	3 × 10^−6^	$C_{13}$	−0.0010
	$C_{23}$	0.0085	$C_{23}$	−0.0010
	$C_{66}$	0.0042	$C_{66}$	−0.0483
LHY2013	*a*_1_	0.9230	*a*_1_	−1.7729
	*a*_2_	1.1087	*a*_2_	0.2303
	*a*_3_	1.1223	*a*_3_	−1.5438
	*a*_4_	1.0992	*a*_4_	−0.8316
	*a*_5_	1.0218	*a*_5_	−0.0958
	*a*_6_	0.9753	*a*_6_	−1.7788
	*a*_7_	0.9160	*a*_7_	0.9472
	*a*_8_	1.2183	*a_8_*	−1.2773
	*h_x_*	−0.0134	*h_x_*	−0.0125
	*h_y_*	−0.0006	*h_y_*	0.0096
Hu & Yoon2021	*μ*	0.0047	*μ*	−0.0097
	$C_{11}$	−0.0267	$C_{11}$	−0.0057
	$C_{22}$	−0.0087	$C_{22}$	0.0263
	$C_{66}$	−24.6485	$C_{66}$	14.3624
	*E*	1.1158	*E*	1.0325
	*F*	1.0323	*F*	0.9847
	*G*	1.0055	*G*	1.0560
A-Eyld2000-2d	$C_{11}^{\prime }$	0.0626	$C_{11}^{\prime }$	−0.9972
	$C_{12}^{\prime }$	1.1393	$C_{12}^{\prime }$	0.0456
	$C_{21}^{\prime }$	1.0457	$C_{21}^{\prime }$	−0.0092
	$C_{22}^{\prime }$	−0.0548	$C_{22}^{\prime }$	−1.1091
	$C_{66}^{\prime }$	0.2116	$C_{66}^{\prime }$	1.0798
	$C_{11}^{\prime \prime }$	0.0605	$C_{11}^{\prime \prime }$	1.0682
	$C_{12}^{\prime \prime }$	−0.7675	$C_{12}^{\prime \prime }$	0.0736
	$C_{22}^{\prime \prime }$	−0.4126	$C_{22}^{\prime \prime }$	−1.0055
	$C_{66}^{\prime \prime }$	1.4650	$C_{66}^{\prime \prime }$	1.2479
	*h_x_*	−0.0150	*h_x_*	−0.0101
	*h_y_*	0.0010	*h_y_*	−0.0049
	*m*	11.2927	*m*	8.4664

**Table A3:** Anisotropy parameters of DP980 and QP980 calculated by different yield criteria.

Yield Criteria	DP980		QP980	
S-Y2004	*α*_1_	7 × 10^−6^	*α*_1_	−5 × 10^−6^
	*α*_2_	1 × 10^−5^	*α*_2_	−6 × 10^−6^
	*λ_y_*_1_	0.9860	*λ_y_*_1_	1.0091
	*λ_y_*_2_	0.9549	*λ_y_*_2_	0.9458
	*ν_y_*	0.5138	*ν_y_*	0.4805
	*ρ_y_*	1.4520	*ρ_y_*	1.4455
CPB06	$C_{11}$	0.0019	$C_{11}$	−0.0005
	$C_{22}$	−0.0036	$C_{22}$	0.0028
	$C_{33}$	−0.0029	$C_{33}$	0.0238
	$C_{12}$	−0.0027	$C_{12}$	0.0241
	$C_{13}$	−0.0034	$C_{13}$	0.0037
	$C_{23}$	0.0016	$C_{23}$	−0.0017
	$C_{66}$	−0.0013	$C_{66}$	−0.0257
LHY2013	*a*_1_	−1.6343	*a*_1_	0.0185
	*a*_2_	1.5577	*a*_2_	−1.6411
	*a*_3_	1.6999	*a*_3_	−1.7365
	*a*_4_	0.0370	*a*_4_	−0.0551
	*a*_5_	0.0027	*a*_5_	0.8565
	*a*_6_	1.6425	*a*_6_	1.6198
	*a*_7_	0.9453	*a*_7_	0.6292
	*a*_8_	0.8113	*a*_8_	1.6660
	*h_x_*	0.0071	*h_x_*	−0.0045
	*h_y_*	0.0098	*h_y_*	−0.0060
Hu & Yoon2021	*μ*	0.0028	*μ*	−0.0032
	$C_{11}$	0.0065	$C_{11}$	−0.0019
	$C_{22}$	0.0103	$C_{22}$	−0.0039
	$C_{66}$	8.3327	$C_{66}$	21.4960
	*E*	0.9684	*E*	0.9373
	*F*	0.9594	*F*	0.9729
	*G*	0.9687	*G*	0.9686
A-Eyld2000-2d	$C_{11}^{\prime }$	0.0030	$C_{11}^{\prime }$	−0.8073
	$C_{12}^{\prime }$	−1.0565	$C_{12}^{\prime }$	0.6162
	$C_{21}^{\prime }$	−1.1262	$C_{21}^{\prime }$	1.2326
	$C_{22}^{\prime }$	−0.0031	$C_{22}^{\prime }$	0.6194
	$C_{66}^{\prime }$	1.1000	$C_{66}^{\prime }$	1.2455
	$C_{11}^{\prime \prime }$	−0.0219	$C_{11}^{\prime \prime }$	0.2957
	$C_{12}^{\prime \prime }$	−0.1449	$C_{12}^{\prime \prime }$	0.7318
	$C_{22}^{\prime \prime }$	−0.7115	$C_{22}^{\prime \prime }$	0.1844
	$C_{66}^{\prime \prime }$	−0.9697	$C_{66}^{\prime \prime }$	−0.1656
	*h_x_*	0.0084	*h_x_*	−0.0047
	*h_y_*	0.0061	*h_y_*	−0.0060
	*m*	5.4945	*m*	3.4011

## References

[1] Hou Y., Myung D., Park J.K., Min J., Lee H.-R., El-Aty A.A., Lee M.-G. (2023). A review of characterization and modelling approaches for sheet metal forming of lightweight metallic materials. Materials.

[2] Wang J., Han M., Zhang C., Rayhan H.M.A., Li X., Lou Y. (2023). Anisotropic hardening of TRIP780 steel sheet: Experiments and analytical modeling. Materials.

[3] Han G., He J., Li S. (2022). Simple shear deformation of sheet metals: Finite strain perturbation analysis and high-resolution quasi-in-situ strain measurement. Int. J. Plast..

[4] Liu X., He J., Huang S.Y. (2023). Mechanistically informed artificial neural network model for discovering anisotropic path-dependent plasticity of metals. Mater. Des..

[5] He J., Guo C., Li W.K. (2023). Understanding the helicoidal damage behavior of bio-inspired laminates by conducting multiscale concurrent simulation and experimental analysis. Compos. Struct..

[6] Guo C., He J. (2022). Multi-scale concurrent analysis for bio-inspired helicoidal CFRP laminates and experimental investigation. Compos. Struct..

[7] Brosius A., Küsters N., Lenzen M. (2018). New method for stress determination based on digital image correlation data. CIRP Ann. -Manuf. Technol..

[8] Lou Y., Zhang C., Zhang S., Yoon J.W. (2022). A general yield function with differential and anisotropic hardening for strength modelling under various stress states with non-associated flow rule. Int. J. Plast..

[9] Hill R. (1948). A theory of the yielding and plastic flow of anisotropic metals. Proc. R. Soc. London. Ser. A Math. Phys. Sci..

[10] Yoshida F., Hamasaki H., Uemori T. (2013). A user-friendly 3D yield function to describe anisotropy of steel sheets. Int. J. Plast..

[11] Barlat F., Lian K. (1989). Plastic behavior and stretchability of sheet metals. Part I: A yield function for orthotropic sheets under plane stress conditions. Int. J. Plast..

[12] Barlat F., Becker R.C., Hayashida Y., Maeda Y., Yanagawa M., Chung K., Brem J.C., Lege D.J., Matsui K., Murtha S.J. (1997). Yielding description for solution strengthened aluminum alloys. Int. J. Plast..

[13] Barlat F., Maeda Y., Chung K., Yanagawa M., Brem J.C., Hayashida Y., Lege D.J., Matsui K., Murtha S.J., Hattori S. (1997). Yield function development for aluminum alloy sheets. J. Mech. Phys. Solids..

[14] Barlat F., Brem J.C., Yoon J.W., Chung K., Dick R.E., Lege D.J., Pourboghrat F., Choi S.-H., Chu E. (2003). Plane stress yield function for aluminum alloy sheets-part 1: Theory. Int. J. Plast..

[15] Barlat F., Aretz H., Yoon J.W., Karabin M.E., Brem J.C., Dick R.E. (2005). Linear transfomation-based anisotropic yield functions. Int. J. Plast..

[16] Banabic D., Comsa D.S., Sester M., Selig M., Kubil W., Mattiasson K., Sigvant M. Influence of constitutive equations on the accuracy of prediction in sheet metal forming simulation. Proceedings of the NUMISHEET.

[17] Cazacu O., Barlat F. (2001). Generalization of Drucker’s yield criterion to orthotropy. Math. Mech. Solids.

[18] Lou Y., Yoon J.W. (2018). Anisotropic yield function based on stress invariants for BCC and FCC metals and its extension to ductile fracture criterion. Int. J. Plast..

[19] Khalfallah A., Alves J.L., Oliveira M.C., Menezes L.F. (2015). Influence of the characteristics of the experimental data set used to identify anisotropy parameters. Simul. Model. Pract. Th..

[20] Khalfallah A., Oliveira M.C., Alves J.L., Menezes L.F. (2020). Constitutive parameter identification of CB2001 yield function and its experimental verification using tube hydroforming tests. Int. J. Mech. Sci..

[21] Lou Y., Zhang S., Yoon J.W. (2019). A reduced Yld2004 function for modeling of anisotropic plastic deformation of metals under triaxial loading. Int. J. Mech. Sci..

[22] Lee E.-H., Stoughton T.B., Yoon J.W. (2017). A yield criterion through coupling of quadratic and non-quadratic functions for anisotropic hardening with non-associated flow rule. Int. J. Plast..

[23] Hu Q., Yoon J.W., Manopulo N., Hora P. (2021). A coupled yield criterion for anisotropic hardening with analytical description under associated flow rule: Modeling and validation. Int. J. Plast..

[24] Chen Z., Wang Y., Lou Y. (2022). User-friendly anisotropic hardening function with non-associated flow rule under the proportional loadings for BCC and FCC metals. Mech. Mater..

[25] Hou Y., Min J., Lin J., Lee M.-G. (2022). Modeling stress anisotropy, strength differential, and anisotropic hardening by coupling quadratic and stress-invariant-based yield functions under non-associated flow rule. Mech Mater..

[26] Spitzig W.A., Sober R.J., Richmond O. (1975). Pressure dependence of yielding and associated volume expansion in tempered martensite. Acta. Metall..

[27] Spitzig W.A., Richmond O. (1984). The effect of pressure on the flow stress of metals. Acta. Metall..

[28] Stoughton T.B., Yoon J.W. (2004). A pressure-sensitive yield criterion under a non-associated flow rule for sheet metal forming. Int. J. Plast..

[29] Cazacu O., Barlat F. (2004). A criterion for description of anisotropy and yield differential effects in pressure-insensitive metals. Int. J. Plast..

[30] Cazacu O., Plunkett B., Barlat F. (2006). Orthotropic yield criterion for hexagonal closed packed metals. Int. J. Plast..

[31] Plunkett B., Cazacu O., Barlat F. (2008). Orthotropic yield criteria for description of the anisotropy in tension and compression of sheet metals. Int. J. Plast..

[32] Li Z., Yang H., Liu J., Liu F. (2022). An improved yield criterion characterizing the anisotropic and tension-compression asymmetric behavior of magnesium alloy. J. Magnes. Alloys.

[33] Khan A.S., Yu S., Liu H. (2012). Deformation induced anisotropic responses of Ti-6Al-4V alloy Part II: A strain rate and temperature dependent anisotropic yield criterion. Int. J. Plast..

[34] Lou Y., Huh H., Yoon J.W. (2013). Consideration of strength differential effect in sheet metals with symmetric yield functions. Int. J. Mech. Sci..

[35] Yoon J.W., Lou Y., Yoon J., Glazoff M.V. (2014). Asymmetric yield function based on the stress invariants for pressure sensitive metals. Int. J. Plast..

[36] Lou Y., Zhang S., Yoon J.W. (2020). Strength modeling of sheet metals from shear to plane strain tension. Int. J. Plast..

[37] Hou Y., Min J., Guo N., Shen Y., Lin J. (2021). Evolving asymmetric yield surfaces of quenching and partitioning steels: Characterization and modeling. J. Mater. Process. Technol..

[38] Hou Y., Min J., Stoughton T.B., Lin J., Carsley J.E., Carlson B.E. (2020). A non-quadratic pressure-sensitive constitutive model under non-associated flow rule with anisotropic hardening: Modeling and validation. Int. J. Plast..

[39] Hou Y., Min J., Lee M.-G. (2023). Non-associated and non-quadratic characteristics in plastic anisotropy of automotive lightweight sheet metals. Automot. Innov..

[40] Hu Q., Yoon J.W. (2021). Analytical description of an asymmetric yield function (Yoon2014) by considering anisotropic hardening under non-associated flow rule. Int. J. Plast..

[41] Hu Q., Chen J., Yoon J.W. (2022). A new asymmetric yield criterion based on Yld2000-2d under both associated and non-associated flow rules: Modeling and validation. Mech. Mater..

[42] Lou Y., Yoon J.W. (2023). Lode-dependent anisotropic-asymmetric yield function for isotropic and anisotropic hardening of pressure-insensitive materials. Part I: Quadratic function under non-associated flow rule. Int. J. Plast..

[43] Vegter H., Abspoel M., Mulder J. (2019). A plane stress yield surface using Bézier curve interpolation in two directions. IOP Conf. Ser. Mater. Sci. Eng..

[44] Du K., Huang S., Shi M., Li L., Huang H., Zhang S., Zheng W., Yuan X. (2021). Effects of biaxial tensile mechanical properties and non-integer exponent on description accuracy of anisotropic yield behavior. Mater. Des..

[45] Hou Y., Du K., El-Aty A.A., Lee M.-G., Min J. (2022). Plastic anisotropy of sheet metals under plane strain loading: A novel non-associated constitutive model based on fourth-order polynomial functions. Mater. Des..

[46] Zhang K., He Z., Zheng K., Yuan S. (2020). Experimental verification of anisotropic constitutive models under tension-tension and tension-compression stress states. Int. J. Mech. Sci..

[47] He Z., Zhang K., Zhu H., Lin Y., Fu M.W., Yuan S. (2022). An anisotropic constitutive model for forming of aluminum tubes under both biaxial tension and pure shear stress states. Int. J. Plast..

[48] Hu Q., Yoon J.W., Chen J. (2023). Analytically described polynomial yield criterion by considering both plane strain and pure shear states. Int. J. Plast..

[49] Hou Y., Min J., El-Aty A.A., Han H.N., Lee M.-G. (2023). A new anisotropic-asymmetric yield criterion covering wider stress states in sheet metal forming. Int. J. Plast..

[50] Du K., Huang S., Hou Y., Wang H., Wang Y., Zheng W., Yuan X. (2023). Characterization of the asymmetric evolving yield and flow of 6016-T4 aluminum alloy and DP490 steel. J. Mater. Sci. Technol..

[51] Du K., Huang S., Li X., Wang H., Zheng W., Yuan X. (2022). Evolution of yield behavior for AA6016-T4 and DP490—Towards a systematic evaluation strategy for material models. Int. J. Plast..

[52] Du K., Ren Y., Hou Y., Li X., Chen S., Sun L., Zuo X., Yuan X. (2023). Parameters identification strategy of yield criterion for accurately predicting anisotropic behavior under near plane strain loading. Chin. Mech. Eng..

[53] Stoughton T.B., Yoon J.W. (2009). Anisotropic hardening and non-associated flow in proportional loading of sheet metals. Int. J. Plast..

[54] Du K., Huang S., Wang H., Yu F., Pan L., Huang H., Zheng W., Yuan X. (2022). Effect of different yield criteria and material parameter identification methods on the description accuracy of the anisotropic behavior of 5182-O aluminum alloy. J. Mater. Eng. Perform..

